# Integrating Genome-Wide Association Study (GWAS) and Marker-Assisted Selection for Enhanced Predictive Performance of Soybean Cold Tolerance

**DOI:** 10.3390/ijms27010165

**Published:** 2025-12-23

**Authors:** Yongguo Xue, Xiaofei Tang, Xiaoyue Zhu, Ruixin Zhang, Yubo Yao, Dan Cao, Wenjin He, Qi Liu, Xiaoyan Luan, Yongjun Shu, Xinlei Liu

**Affiliations:** 1 Soybean Research Institute, Heilongjiang Academy of Agricultural Sciences, Harbin 150086, China; xyg81@126.com (Y.X.); xftang@126.com (X.T.); yaoyubo2009@aliyun.com (Y.Y.); caodan825@163.com (D.C.); nkydds@126.com (W.H.); liuqi0316@163.com (Q.L.); luanxiaoyan1201@163.com (X.L.); 2 Key Laboratory of Molecular Cytogenetics and Genetic Breeding of Heilongjiang Province, College of Life Science and Technology, Harbin Normal University, Harbin 150025, China; zhuxiaoyue2001@126.com (X.Z.); zrxin2001@126.com (R.Z.)

**Keywords:** genetic evaluation, genome selection (GS), population structure, machine learning, Bayesian method

## Abstract

Soybean (*Glycine max* (L.) Merr.), as a crucial source of oil and protein globally, is widely cultivated in many countries. Low-temperature stress has become one of the major environmental factors affecting soybean production, especially in colder regions, making the improvement of cold tolerance traits in soybean a key breeding objective. This study integrates Genome-Wide Association Studies (GWAS) and Marker-Assisted Selection (MAS) to enhance the predictive performance of soybean cold tolerance traits. First, three GWAS methods—Fast3VmrMLM, fastGWA, and FarmCPU—were used to analyze soybean cold tolerance traits, and significant SNP markers were identified. Principal Component Analysis (PCA) was employed to reveal genetic differences among various soybean germplasm. Then, based on the identified SNP markers, multiple Genomic Selection (GS) models, such as GBLUP, BayesA, BayesB, BayesC, BL, and BRR, were used for prediction to evaluate the contribution of genetic effects to phenotypic variation. The results showed that the markers selected through GWAS significantly improved the prediction accuracy of genomic selection, especially with the Fast3VmrMLM and FarmCPU methods in larger datasets. Finally, Gene Ontology (GO) analysis was performed to further identify candidate genes associated with cold tolerance traits and their biological functions, providing theoretical support for molecular breeding of cold-tolerant soybean varieties.

## 1. Introduction

Soybean (*Glycine max* (L.) Merr.), a crucial source of oil and protein globally, is widely cultivated in numerous countries, particularly in major producing regions such as China, the United States, and Brazil [1]. With the intensification of global climate change, the frequency of extreme weather events has risen, with low-temperature cold stress emerging as one of the primary environmental factors affecting soybean production [2]. Cold stress not only directly impacts seed germination and seedling emergence but can also hinder early growth, slow plant development, and ultimately compromise both yield and quality [3]. Specifically, empirical studies have quantified these impacts, reporting that cold stress can lead to yield losses of approximately 24% in soybeans, resulting in substantial economic deficits for agricultural production [4]. This issue is particularly pronounced in cold regions such as Northeast China, where springtime chilling stress (characterized by soil temperatures frequently dropping below 6 °C for prolonged periods) significantly affects soybean crops. Unlike freezing stress (<0 °C), this chilling stress (0–15 °C) primarily hinders seed imbibition and early metabolic activation without causing intracellular ice formation. This stress is most damaging during the critical germination and seedling emergence stages, leading to poor stand establishment. Consequently, enhancing cold tolerance, especially during the germination phase, has become a key focus in efforts to improve soybean production in these colder regions [5].

In soybean cold tolerance research, traditional methods for assessing cold resistance primarily rely on phenotypic screening and artificial cold stress treatments. While these approaches have provided crucial data for the selection of cold-tolerant varieties, their accuracy and efficiency remain significantly limited [1]. For instance, previous research has indicated that the coefficient of variation (CV) for cold tolerance traits (such as relative germination rate) can range from 0.94 to 1.33, highlighting the substantial instability caused by environmental noise [6]. On one hand, phenotypic screening is highly influenced by environmental factors, making it difficult to accurately reflect the relationship between genotype and phenotype [7]. On the other hand, cold tolerance is a complex trait controlled by multiple genes and influenced by various environmental factors [8]. Consequently, the precise identification of genes associated with cold tolerance and the effective prediction of this trait have become critical challenges in current soybean cold tolerance research.

With the advancement of high-throughput genomic technologies, Genome-Wide Association Studies (GWAS) have become one of the core techniques for uncovering the genetic basis of complex traits in crops. Through GWAS, researchers can analyze the associations between genotype and phenotype data to identify SNP markers linked to target traits, thereby providing a foundation for Marker-Assisted Selection (MAS). Deshmukh, R. et al. (2014) [9] utilized GWAS to uncover several key genetic loci associated with stress tolerance in soybean, offering a theoretical basis for the improvement of traits such as cold tolerance and disease resistance. Despite the significant progress made by GWAS in the genetic analysis of crop traits, relying solely on GWAS for traits with complex genetic architectures, such as cold tolerance—characterized by multi-gene control and significant environmental influence—remains limited in terms of accuracy. In recent years, Genome Selection (GS) has gradually been introduced as an innovative technique in crop breeding. GS leverages genotype and phenotype data to establish regression models, accelerating the breeding process by predicting the genetic effects of traits. Compared to traditional phenotypic selection methods, GS allows for direct trait prediction based on genotype data, thereby circumventing the interference of complex environmental factors and phenotypic data. Early GS studies largely adopted the mixed linear model–based genomic selection approach (GBLUP) proposed by Meuwissen et al. (2001) [10]. Although GBLUP has performed robustly in many crops, its reliance on the infinitesimal model assumption—namely, that all marker effects follow the same normal distribution with very small variance—limits its ability to capture major-effect loci. To address this limitation, Bayesian approaches such as BayesA and BayesB, as well as machine-learning algorithms including support vector machines (SVM) and random forests, have been developed and widely applied in crop breeding [11,12,13]. Compared with linear models, Bayesian methods allow markers to have different variance distributions, enabling more accurate detection of major-effect QTL controlling the trait. Machine-learning algorithms, in turn, offer distinct advantages in capturing complex nonlinear relationships, particularly for genotype-by-environment interactions and non-additive genetic effects (e.g., epistasis). Existing studies have already demonstrated the substantial potential of optimizing model selection. For example, in an alfalfa study, the mean prediction accuracy based on a genome-wide LD-pruned SNP set was only 0.11–0.18, whereas using GWAS-derived significant markers substantially increased PA to 0.70–0.80 [14]. Mechanistically, GS relies on the pervasive Linkage Disequilibrium (LD) between molecular markers and Quantitative Trait Loci (QTLs) throughout the genome. Unlike phenotypic selection, which is often confounded by environmental variability, GS captures the total additive genetic variance—including contributions from minor-effect loci—allowing for the calculation of Genomic Estimated Breeding Values (GEBVs) that are less biased by environmental noise. However, integrating GWAS with GS involves a trade-off. It is widely recognized that for highly polygenic traits, using exclusively GWAS-significant markers can reduce prediction accuracy due to the exclusion of small-effect loci—a phenomenon known as “missing heritability”. Therefore, determining the optimal balance between removing noise (irrelevant markers) and retaining polygenic signals is a critical challenge in refining prediction models. However, although single models or specific strategies have made progress in some crops, most complex traits are controlled by many loci with small effects. In addition, LD decay and imperfect concordance between markers and causal variants mean that, under the “all-marker, high-dimensional (*p* >> n)” setting, models are prone to introducing noise and overfitting. Therefore, for soybean cold tolerance, a key unresolved challenge is how to organically integrate GWAS-based discovery with GS modeling and to leverage the complementary strengths of different algorithms to break through the current bottleneck in predictive accuracy [3,15].

In soybean cold tolerance research, the integration of Genome-Wide Association Studies and Genome Selection approaches, utilizing traditional models, Bayesian models, and machine learning techniques, offers a promising new avenue for the precise prediction of cold tolerance traits. Traditional GWAS methods are typically used to identify SNP markers significantly associated with phenotypes, while genomic selection (GS) focuses on using these SNP markers for accurate prediction of complex traits. Combining both approaches can compensate for the limitations of each method, especially when dealing with complex traits and large-scale genomic data. Traditional linear models may not fully capture the intricate gene-phenotype relationships, but the integration of GWAS and GS optimizes this process through multiple algorithms, making it better equipped to handle high-dimensional data and multi-gene effects, thereby improving prediction accuracy and model stability. Cold tolerance in soybeans, as a complex trait, is influenced by multiple genes and gene-environment interactions. By combining GWAS with genomic selection, it is possible to more accurately predict cold tolerance performance in soybeans with different genetic backgrounds based on the screening of genetic markers related to cold tolerance. This strategy goes beyond the traditional single-function gene discovery approach, offering richer biological insights for precise breeding, and thus providing new ideas and directions for agricultural breeding.

Based on this concept, the present study aims to enhance the prediction accuracy of soybean cold tolerance traits through the synergistic application of GWAS and GS. Specifically, we first employed three GWAS methods—Fast3VmrMLM, fastGWA, and FarmCPU—to conduct genotype-phenotype association analysis on 156 soybean germplasm resources from China and Europe. Fast3VmrMLM can efficiently handle large-scale data, making it particularly suitable for analyzing complex gene-phenotype associations. BOLT-LMM, as an efficient linear mixed model, effectively controls the effects of population structure and genetic relationships, thereby improving the accuracy of genotype-phenotype association analysis. FarmCPU combines fixed and random effects, using iterative optimization to effectively control false positives, providing more stable and reliable analysis results. By utilizing these methods, we were able to identify SNP markers significantly associated with cold tolerance, providing a reliable foundation for subsequent genomic selection analysis. This integrated approach combining GWAS and GS offers a novel strategy for genomic selection of soybean cold tolerance traits and establishes a solid theoretical foundation for precision breeding of cold-tolerant soybeans. With the continuous advancement of genomics and machine learning technologies, genomic selection is expected to be more widely applied in soybean and other crop breeding, thereby promoting the sustainable development of agricultural production.

## 2. Results

### 2.1. Phenotypic and SNP Distribution

**Figure 1:**
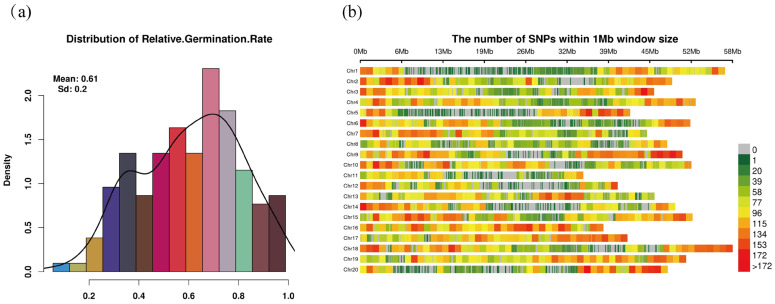
Distribution of Relative Germination Rate and SNP Density. (**a**) Distribution of relative germination rate across soybean germplasm. The histogram displays the frequency distribution of relative germination rates, with the mean (0.61) and standard deviation (0.2) indicated. The curve represents the fitted density distribution. (**b**) SNP density distribution across the soybean genome. The heatmap shows the number of SNPs within 1 Mb sliding windows for each chromosome, with color intensity representing the number of SNPs (ranging from 0 to >172). The SNP distribution across the chromosomes highlights regions of high density (red) and low density (green), providing insights into the genomic regions with the highest genetic variation.

Statistical analysis of the relative germination rates under low-temperature stress was conducted for various soybean germplasm resources. As shown in Figure 1a, the distribution of relative germination rates approximates a normal distribution, with a mean of 0.61 and a standard deviation of 0.20. The distribution indicates that the majority of germplasm resources exhibit relative germination rates between 0.5 and 0.7, suggesting moderate cold tolerance under low-temperature stress. Despite the overall normal distribution trend, the left skewness (skewness coefficient = −0.23) of the data indicates that some germplasm resources have relatively low germination rates, reflecting weaker cold tolerance under cold stress conditions. These results highlight significant genetic variation in cold tolerance among soybean germplasm, with most displaying moderate germination ability under low-temperature stress. This provides foundational data for the subsequent screening of cold tolerance traits and genomic selection.

To further explore genetic variation in the soybean genome, SNP density distributions were calculated and displayed for each chromosome. As shown in Figure 1b, SNP density was computed using a 1 Mb sliding window for each chromosome. The results reveal significant regional variation in SNP distribution, with SNP density varying across different chromosomal regions. Based on the color scale, red regions indicate higher SNP density, whereas green and yellow regions indicate lower SNP density; SNP density ranges from 0 to 172 SNPs/Mb. The distribution of SNP density on chromosomes not only reflects the structural characteristics of the genome but may also be linked to the genetic mechanisms underlying cold tolerance traits, providing valuable insights for subsequent analyses.

### 2.2. PCA Results

**Figure 2:**
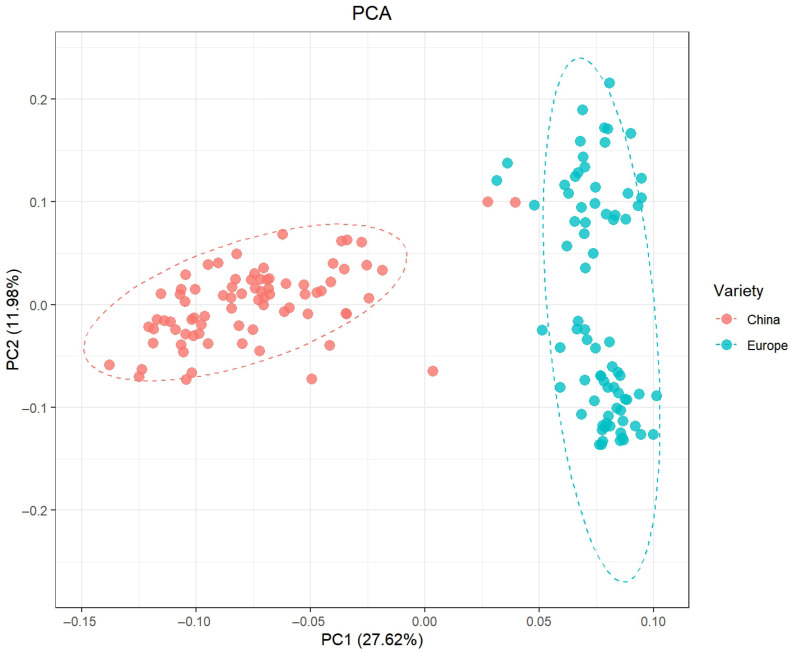
Principal Component Analysis (PCA) of Soybean Germplasm. PCA plot showing the distribution of soybean germplasm based on the first two principal components (PC1 and PC2). Each point represents a single soybean sample, with the *x*-axis representing PC1 and the *y*-axis representing PC2. The plot reveals clear separation of the germplasm into two distinct clusters, indicating significant genetic variation among the soybean samples.

As shown in Figure 2, Principal Component Analysis (PCA) based on the first two principal components (PC1 and PC2) reveals the genetic variation distribution of soybean germplasm under low-temperature stress. The *x*-axis, PC1, explains 27.62% of the total variation, while the *y*-axis, PC2, explains 11.98% of the variation. The scatter plot shows significant separation of the samples along the PC1 axis, forming two distinct clusters: the China-origin (red dots) varieties are primarily distributed in the negative half of the PC1 axis, with their distribution along PC2 being relatively concentrated; the Europe-origin (blue dots) varieties are mainly found in the positive half of the PC1 axis, showing greater dispersion along the PC2 axis (more spread vertically). This significant geographic-based clustering suggests that soybean germplasm from China and Europe exhibit distinct population structure differences in response to low-temperature stress, closely related to their unique genetic backgrounds. This result not only visually demonstrates the genetic diversity of soybean germplasm in terms of cold tolerance but also provides a reliable statistical basis for the subsequent localization and screening of cold tolerance-related genes in different subgroups.

### 2.3. PVE Calculation Results

**Figure 3:**
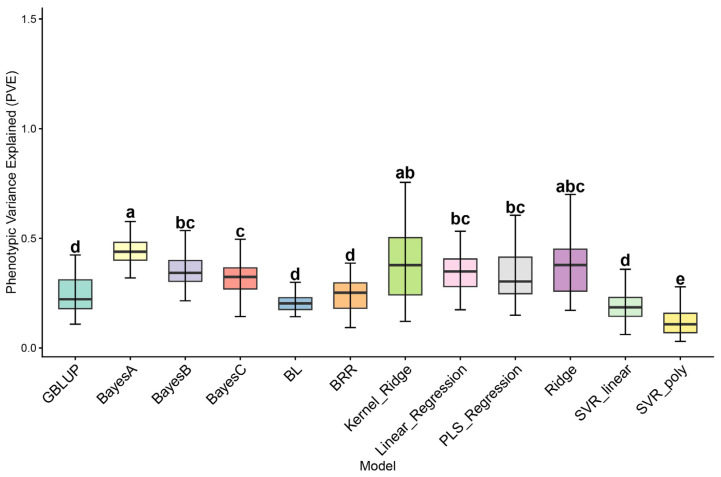
PVE (Phenotypic Variance Explained) by Different Genomic Selection Models. Bar plot illustrating the Phenotypic Variance Explained (PVE) by different genomic selection models. The *x*-axis represents the models used (GBLUP, BayesA, BayesB, BayesC, BL, BRR, Kernel Ridge, Linear Regression, PLS Regression, Ridge, SVR_linear, and SVR_poly), while the *y*-axis represents the PVE value for each model. The plot shows that the Bayes series models (BayesA, BayesB) and traditional models such as GBLUP and BRR generally outperform other models, with SVR models (SVR_linear, SVR_poly) showing much lower PVE values. Different lowercase letters (a–e) above the boxes indicate statistically significant differences between models (*p* < 0.05); models sharing the same letter are not significantly different.

To evaluate the explanatory power of different genomic selection models in predicting cold tolerance traits, the Phenotypic Variance Explained (PVE) by each model was calculated. The PVE value measures the contribution of each model to the genetic variation in soybean cold tolerance traits. By assessing the explanatory capacity of various models for cold tolerance traits, this provides important insights for subsequent genomic selection efforts. As shown in Figure 3, the PVE values of different genomic selection models in predicting cold tolerance traits are presented. The PVE values reflect the ability of each model to explain the genetic variation in soybean cold tolerance traits. Evaluation of multiple regression and machine learning models revealed that the BayesA model performed the best among all models, with a PVE value of 0.46, indicating that this model effectively explains a large proportion of the variation in cold tolerance traits. Following closely are the BayesB, BayesC, and BL models, which exhibited similar PVE values ranging from 0.35 to 0.40, indicating strong performance in predicting cold tolerance traits. In contrast, the GBLUP model yielded a lower PVE value of 0.28 compared to the Bayesian models. This reduced performance is likely attributable to the ‘infinitesimal model’ assumption of GBLUP, which presumes that all markers share a common variance and have small effects. Consequently, GBLUP may have failed to adequately capture the large-effect loci (major QTLs) associated with cold tolerance that were identified in our GWAS analysis, whereas Bayesian models (e.g., BayesA) can accommodate such major effects by allowing for marker-specific variances. The Kernel Ridge and PLS Regression models achieved PVE values of 0.32 and 0.30, respectively, showing some predictive ability but still lower than the Bayesian regression models. The SVR_linear and SVR_poly models had significantly lower PVE values, indicating poor performance in predicting cold tolerance traits.

### 2.4. GWAS Analysis

**Figure 4:**
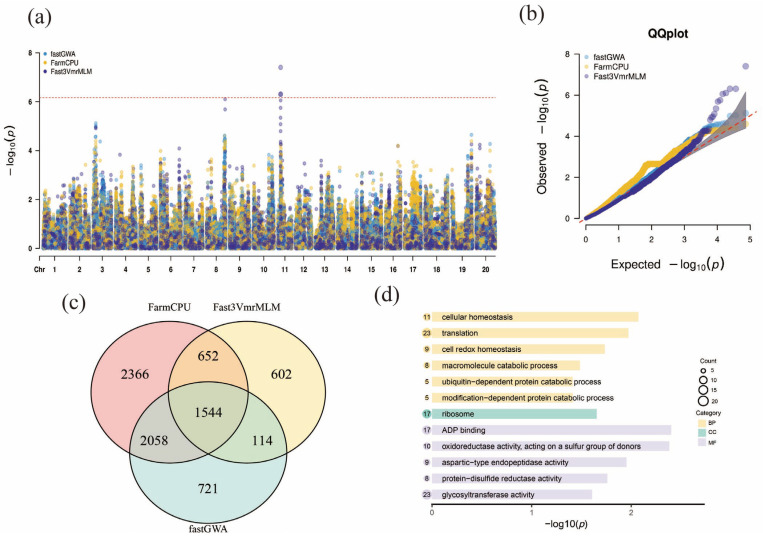
GWAS Results and Functional Analysis of Soybean Cold Tolerance. (**a**) Manhattan plot showing the results of GWAS conducted using three methods: fastGWA (blue), FarmCPU (yellow), and Fast3VmrMLM (purple). The −log10(*p*) values for each SNP are plotted against their chromosomal positions, with the red dashed line indicating the significance threshold. (**b**) QQ plot comparing the observed −log10(*p*) values against the expected values for each of the three GWAS methods. The plot illustrates the distribution of SNPs with fastGWA, FarmCPU, and Fast3VmrMLM showing varying degrees of deviation from the expected distribution. The red dashed line indicates the theoretical null distribution; deviations from this line suggest potential associations. (**c**) Venn diagram showing the overlap of significant SNPs identified by the three GWAS methods. The numbers indicate the number of SNPs unique to each method (FarmCPU: 2366, Fast3VmrMLM: 2058, fastGWA: 721) and the shared SNPs (652). (**d**) Gene Ontology (GO) enrichment analysis of the genes associated with significant SNPs from the GWAS results. The bar chart shows the top enriched biological processes (BP), cellular components (CC), and molecular functions (MF), with the size of the circles representing the count of enriched genes in each category. The processes include cellular homeostasis, translation, and redox homeostasis.

This study employed three different GWAS methods (fastGWA, FarmCPU, and Fast3VmrMLM) to identify significant SNP loci associated with soybean cold tolerance traits, with a strict Bonferroni correction applied to the significance threshold to control the family-wise error rate (FWER = 0.05). To visually present the results, Manhattan plots, QQ plots, and Venn diagrams were used, and Gene Ontology (GO) enrichment analysis was further conducted. The Manhattan plot (Figure 4a) illustrates the association strength between SNP loci in the soybean genome and cold tolerance traits. Each point represents an SNP, with the *y*-axis indicating its significance (−log10(*p*) value) and the *x*-axis representing the chromosomal position. Points in different colors correspond to the three GWAS methods (blue for fastGWA, yellow for FarmCPU, and purple for Fast3VmrMLM). The plot reveals significant SNP clustering in several chromosomal regions, particularly in regions 8 and 11, suggesting that these areas are closely associated with the genetic variation in cold tolerance traits. The red dashed line represents the significance threshold, with points above this line considered significant. These significant SNPs provide potential target regions for further investigation. The QQ plot (Figure 4b) further compares the observed −log10(*p*) values with the expected values. The observed −log10(*p*) values aligned closely with the expected values for the vast majority of markers, indicating that population stratification was effectively controlled and there was no evidence of genomic inflation or deflation. The upward deviation of the markers at the tail end of the distribution signifies true genotype-phenotype associations rather than spurious signals arising from systematic errors.

The Venn diagram (Figure 4c) displays the overlap of significant SNPs identified by the three GWAS methods. The results show that the Fast3VmrMLM and FarmCPU methods identified approximately 1544 common significant SNPs, demonstrating a high level of consistency between these two methods in the genetic analysis of soybean cold tolerance traits. Meanwhile, the fastGWA method independently identified 2366 SNPs, with minimal overlap with the other two methods, possibly reflecting the unique sensitivity of this method to certain cold tolerance-related genes. Finally, the significant loci identified by Fast3VmrMLM were functionally annotated and mapped to nearby genes, as shown in Table 1. The GO enrichment analysis (Figure 4d) revealed the biological processes, molecular functions, and cellular components associated with the SNPs related to cold tolerance traits. In the biological process (BP) category, the most significantly enriched terms included translation (23 genes), cellular homeostasis (11 genes), and cell redox homeostasis (9 genes). The prominent enrichment of translation was highly consistent with the top enriched cellular component (CC) term, ribosome (17 genes). This strongly suggests that, under low-temperature stress, soybean maintains cellular function and homeostasis by optimizing and protecting the protein synthesis machinery (ribosomes), thereby ensuring rapid production and proper folding of stress-responsive proteins. In the molecular function (MF) category, we observed significant enrichment of multiple oxidoreductase activities (e.g., oxidoreductase activity acting on a sulfur group of donors; protein-disulfide reductase activity), as well as ADP binding (17 genes) and glycosyltransferase activity (23 genes). These functions are directly linked to key physiological mechanisms underlying cold-stress responses in plants. Elevated oxidoreductase activities indicate activation of antioxidant defense systems to scavenge cold-induced reactive oxygen species (ROS), whereas glycosyltransferase activity is commonly involved in cell-wall modification and glycosylation of membrane proteins, which are critical for enhancing membrane stability and maintaining membrane lipid fluidity. Taken together, these results suggest that the genetic basis of soybean cold tolerance involves a multilayered defense network, encompassing fine-tuned regulation of metabolism and energy balance (ADP binding), as well as efficient protein synthesis and repair mechanisms and a strong oxidative-stress defense capacity. These enriched biological processes suggest that soybean cold tolerance traits may be closely linked to cellular stress responses, resistance mechanisms, and the regulation of fundamental cellular functions.

In conclusion, this GWAS analysis provides valuable information for the genetic localization and functional study of soybean cold tolerance traits. By comparing different GWAS methods, the strengths and limitations of each method in identifying significant SNPs and explaining genetic variation were evaluated. Furthermore, the GO enrichment analysis offers a theoretical basis for a deeper understanding of the molecular mechanisms underlying cold tolerance traits.

### 2.5. Joint GWAS-GS Analysis

To further evaluate the impact of GWAS screening methods on the prediction accuracy of Genome Selection, this study compared various GWAS methods across different dataset sizes. Specifically, SNP markers identified by the Fast3VmrMLM, fastGWA, and FarmCPU GWAS methods were used for genome selection analysis of soybean cold tolerance traits, with different dataset sizes (500, 1000, 5000, 10,000, and 50,000 markers). By comparing the prediction results of different models based on these selected markers, the study systematically assessed how GWAS methods influence the prediction accuracy of GS.

**Figure 5:**
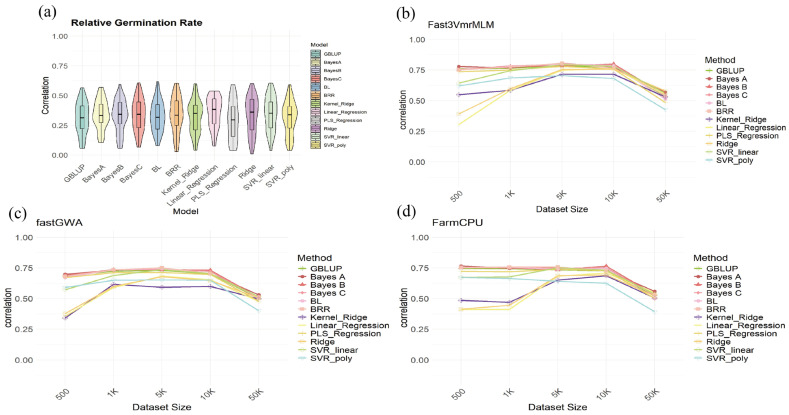
Comparison of Genomic Selection Performance Using Different Models and GWAS-Selected Markers. (**a**) Genomic Selection (GS) with All Markers: Violin plot showing the correlation between predicted and observed relative germination rates for different GS models using all SNP markers. (**b**) GS with Fast3VmrMLM-Selected Markers: Line plot illustrating the correlation for different GS models based on SNP markers selected by the Fast3VmrMLM method, evaluated across various dataset sizes (500, 1K, 5K, 10K, 50K markers). (**c**) GS with fastGWA-Selected Markers: Line plot showing the correlation for different GS models based on SNP markers selected by the fastGWA method, evaluated across varying dataset sizes. (**d**) GS with FarmCPU-Selected Markers: Line plot demonstrating the correlation for different GS models based on SNP markers selected by the FarmCPU method, evaluated across different dataset sizes.

As shown in Figure 5, four subplots are presented, representing the genome selection analysis results for all markers and those selected by the three GWAS methods. Each subplot shows the prediction correlation for different genomic selection models (such as GBLUP, BayesA, BayesB, BayesC, BL, BRR, and SVR) at varying dataset sizes. Figure 5a presents the results of the analysis using all markers. For all markers, the prediction accuracy is approximately between 0 and 0.6, with the mean never exceeding 0.5. The distribution trends for each method are relatively consistent. Next, Figure 5b–d show the prediction accuracy (Pearson r) after marker selection using different methods, where a noticeable improvement to around 0.75 is observed. This represents a substantial increase of approximately 40% compared to the baseline accuracy obtained using all markers. This significant improvement suggests that identifying and prioritizing SNP markers with strong phenotypic associations is crucial for reducing genomic noise, particularly in smaller populations, thereby maximizing the predictive power of the models. Both FarmCPU and Fast3VmrMLM methods show similar trends in this regard. Furthermore, among all the models, Bayesian models consistently demonstrated stable predictions.

Additionally, while more than 70,000 markers were considered, the optimal performance was observed when approximately 10,000 markers were selected. This suggests that too few markers may lead to underutilization, while too many markers could introduce noise from irrelevant information. In summary, the GWAS methods significantly enhanced the prediction accuracy of genome selection models. Specifically, markers selected by the Fast3VmrMLM and FarmCPU methods were able to effectively improve prediction accuracy in larger datasets. This demonstrates that combining GWAS with Genome Selection (GS) methods can significantly improve the prediction accuracy of soybean cold tolerance traits, providing a powerful tool for the development of cold-tolerant soybean varieties in the future.

## 3. Discussion

This study integrates Genome-Wide Association Studies (GWAS) and Genome Selection (GS) methods to explore the genetic mechanisms of soybean cold tolerance traits and evaluate the prediction accuracy using various analytical approaches. The results demonstrate that the combination of GWAS and GS methods not only reveals the genetic variation underlying soybean cold tolerance traits but also significantly improves predictive accuracy. The following discussion will elaborate on these findings from multiple perspectives.

### 3.1. GWAS Results and Significant SNP Markers Associated with Cold Tolerance Traits

The GWAS analysis made significant progress in identifying SNP markers associated with soybean cold tolerance traits. In this study, three GWAS methods—Fast3VmrMLM, fastGWA, and FarmCPU—were used to identify multiple SNP loci associated with cold tolerance traits. Principal Component Analysis (PCA) revealed genetic differences among the different germplasm, further validating the effectiveness of the GWAS methods in uncovering the genetic basis of cold tolerance traits in different soybean germplasm. Specifically, the Fast3VmrMLM method performed well across all models, capturing strong genetic signals, particularly in high-density SNP regions [16]. At the same time, the FarmCPU method demonstrated high consistency in prediction accuracy, which aligns with its ability to effectively control population structure and reduce environmental interference. These results suggest that soybean cold tolerance is a complex trait controlled by multiple genes and is closely related to genetic background.

### 3.2. Analysis of PVE Differences

In this study, the BayesA and KRR models achieved higher phenotypic variance explained, largely because they are better suited to the complex genetic architecture underlying soybean cold tolerance. Cold tolerance often follows an oligogenic or mixed genetic model, in which a few major-effect genes act together with many minor-effect loci. The differences among models can be summarized as follows. Advantages of Bayesian models. Bayesian methods such as BayesA and BayesB, through flexible Bayesian regression frameworks, allow marker-specific variances. This property enables them to better capture and distinguish the effects of minor-effect loci and major-effect QTLs, avoiding excessive shrinkage of small effects and thereby improving predictive accuracy. This mechanism may also help explain the strong performance observed for Fast3VmrMLM in our study, as this method applies machine-learning–based pre-screening of markers, substantially enriching for major and highly associated signals. Limitations of GBLUP. In contrast, the weaker performance of GBLUP (PVE = 0.28) is likely attributable to its infinitesimal-model assumption, namely that all marker effects follow a single normal distribution with a common variance. When a trait is influenced by a small number of major loci, this assumption tends to over-distribute (or over-shrink) large effects, preventing GBLUP from fully capturing the major cold-tolerance loci identified by GWAS and resulting in a lower PVE. Underperformance of SVR. The substantially lower PVE for support vector regression (SVR) is unlikely to reflect a simple inability to handle high-dimensional data. Rather, as a nonparametric kernel-based method, SVR may struggle to fit a robust regression function when the sample size is relatively small (156 accessions in this study) and the trait is governed by the cumulative contributions of many small-effect loci. In addition, SVR can be sensitive to noise points (outliers) and sparsity in high-dimensional genomic data, which may reduce model robustness and ultimately compromise predictive performance.

### 3.3. GWAS Selection Enhances GS Prediction Accuracy

This study made substantial progress in identifying SNP markers associated with soybean cold tolerance. We integrated three GWAS approaches—Fast3VmrMLM, fastGWA, and FarmCPU—to detect multiple cold tolerance–associated SNP loci. First, principal component analysis was used to characterize genetic differences among the tested accessions, and the first three principal components were included as covariates in the GWAS models to effectively correct for population structure. Our results clearly demonstrate that GWAS-preselected markers can significantly improve the predictive accuracy of genomic selection (GS) models. This improvement is not merely a consequence of reduced marker numbers and the resulting computational gains, but reflects an intrinsic enhancement in marker quality. The key strategy lies in coupling biological insight with statistical optimization: GWAS identifies SNPs that are significantly associated with the phenotype, and these SNPs are more likely to be in strong linkage disequilibrium (LD) with causal variants underlying cold tolerance. Consequently, the markers selected by methods such as Fast3VmrMLM can serve as proxy markers that more precisely tag major-effect loci (major QTLs). Compared with sparse, genome-wide marker sets, these enriched subsets transfer more relevant genetic information to GS models. For instance, our data showed that using a subset of ~10,000 Fast3VmrMLM-selected markers achieved a prediction accuracy of 0.78, significantly outperforming the model using all 76,158 markers (r = 0.46). This demonstrates that prioritizing high-confidence loci effectively amplifies the genetic signal while reducing the dimensionality of the data. In addition, using all markers without filtering introduces substantial noise because many loci have negligible or no effect. GWAS-based screening enriches for markers contributing most to phenotypic variance, enabling GS models to focus more effectively on capturing and explaining additive genetic variance. Markers showing low LD with GWAS-positive signals can be interpreted as potential false positives or extremely small-effect loci and are removed during filtering, thereby improving the signal-to-noise ratio—an important driver of better predictive performance.

Notably, the strong performance of Fast3VmrMLM is not incidental; it reflects an ongoing methodological shift in GWAS toward integrating mixed models with machine-learning strategies. By combining the strengths of multi-locus mixed linear models (MLMLM) with iterative, machine-learning–assisted optimization, Fast3VmrMLM can disentangle tightly linked QTLs more effectively than traditional single-locus models while controlling false positive associations. This advanced screening mechanism yields marker subsets that more comprehensively capture the heterogeneous genetic variance underlying cold tolerance. When these high-precision, high–signal-to-noise marker subsets are subsequently used in Bayesian models (e.g., BayesA), the Bayesian advantage in handling sparse signals and capturing major effects is maximized, resulting in a marked increase in predictive accuracy. Collectively, these findings indicate that, for GS of complex traits such as soybean cold tolerance, integrating the statistical power of GWAS with the screening efficiency of machine learning is critical for building robust prediction models and ultimately accelerating molecular breeding.

### 3.4. Limitations of the Study and Future Prospects

Although this study significantly improved the prediction accuracy of soybean cold tolerance traits by integrating GWAS and GS methods, several limitations still exist [17]. First, this study only considered phenotypic data for cold tolerance traits. Future research should focus on developing more robust genotype-by-environment (G × E) prediction models. To this end, integrating a broader range of environmental factors will be necessary to further improve model generalizability. Beyond low-temperature stress per se, subsequent work should prioritize incorporating key meteorological and microenvironmental variables that influence soybean growth and physiology, including diurnal and seasonal temperature fluctuations, humidity, and light intensity and photoperiod. More specifically, soil microenvironmental parameters (e.g., soil temperature dynamics and soil moisture content) should also be considered, as these factors directly govern seed germination and early root development and are critical for achieving high-accuracy prediction under field conditions [18]. For instance, environmental factors such as temperature variations, humidity, and light conditions may have significant impacts on soybean cold tolerance, and including these variables could improve the model’s adaptability and prediction accuracy under different environmental conditions [19,20]. Secondly, the marker selection methods used in this study were primarily focused on the seedling stage of cold tolerance. Future studies could expand to include cold tolerance traits at other growth stages (such as the germination stage and mature plant stage) in genomic selection. This would help to comprehensively assess the genetic mechanisms and adaptability of soybean cold tolerance across different growth stages.

Additionally, the SNP marker density used in the GWAS analysis in this study was relatively low, which limits the comprehensive identification of genes related to cold tolerance in the soybean genome [19,21,22]. With advancements in technology, future research could use higher-density SNP chips or whole-genome sequencing technologies to enhance the ability to identify SNP markers, thereby more precisely locating genes and QTLs (quantitative trait loci) related to cold tolerance [23,24,25,26]. Future research should focus on several key areas: First, by incorporating a broader set of cold tolerance phenotypes, future efforts to further optimize genomic selection models for soybean cold tolerance should not be limited to evaluating root growth rate alone. Instead, they should comprehensively characterize root architectural traits—such as root system configuration, branching density, and root surface area—because these structural attributes directly determine the plant’s efficiency in acquiring water and nutrients under low-temperature stress. Integrating growth and architectural traits would provide more complete phenotypic information, enabling better capture of genotype-by-environment interactions and, consequently, improving both the predictive accuracy and robustness of models for this complex cold tolerance trait. Second, it would be valuable to explore combining other types of molecular markers (such as insertion-deletion markers, structural variations, etc.) with SNP markers for genomic selection to improve prediction accuracy. Lastly, as the soybean genome continues to be refined, genomic selection methods will be more widely applied in soybean and other crop breeding, particularly in the precise breeding of stress-resistant traits, making genomic selection an increasingly important tool in improving crop stress resistance and stability.

## 4. Materials and Methods

### 4.1. Plant Materials

This study utilized a total of 156 soybean germplasm samples, of which 79 were sourced from China, primarily from the northeastern region, all of which are cultivated varieties. The remaining 77 samples were from Europe, representing 10 countries, specifically: 24 from Serbia, 11 from Austria, 10 from Italy, 10 from Switzerland, 5 from France, 4 from Romania, 4 from Poland, 4 from Ukraine, 3 from Hungary, and 2 from Germany. Detailed information on the soybean germplasm resources is provided in Table S1.

### 4.2. Phenotypic Data Collection

The phenotypic data used in this study were primarily sourced from the dataset provided by Han et al. (2022) [6], which were collected through an indoor germination cold tolerance experiment. Specifically, seeds were soaked in a 5% sodium hypochlorite solution for 30 s, followed by two rinses with sterile water. The seeds were then placed in sterilized Petri dishes, with an appropriate amount of distilled water added. Two layers of filter paper were placed at the bottom of each dish, and the seeds were allowed to imbibe at room temperature for 12 h. After imbibition, 600 seeds were selected and divided into two groups: low-temperature treatment and control. Each group had three biological replicates, with 100 seeds per replicate. For the low-temperature treatment group, the seeds were placed in a 6 °C constant-temperature incubator, while the control group was kept in a 25 °C constant-temperature incubator. All groups were kept in the dark, and distilled water was replaced every 3 days to prevent mold growth. The cold tolerance of the seeds was ultimately assessed by calculating the relative germination rate, which served as the indicator for cold tolerance evaluation.

$$\mathrm{Relative}\;\mathrm{Germination}\;\mathrm{Rate}=\frac{\mathrm{Low}\text{-}\mathrm{Temperature}\;\mathrm{Stress}\;\mathrm{Germination}\;\mathrm{Rate}}{\mathrm{Normal}\;\mathrm{Sowing}\;\mathrm{Germination}\;\mathrm{Rate}}\times 100\%$$

Here, the germination rate at 25 °C serves as the denominator (control) to normalize for variations in intrinsic seed viability.

### 4.3. Genomic DNA Extraction and SNP Genotyping

Genomic DNA was extracted using an improved CTAB method [27]. SNP genotyping was performed using the ‘Zhongdouxin No. 1’ soybean SNP chip, developed jointly by the Institute of Crop Sciences, Chinese Academy of Agricultural Sciences, and Compson Biotechnology Co., Ltd. (Beijing, China), resulting in the identification of 158,327 SNP loci. The data were filtered using VCFtools to remove SNP loci with a missing rate greater than 20% and minor allele frequency (MAF) less than 0.05 [28]. The filtered data were then imputed for missing values using the default parameters of Beagle V5.4 software [29]. Ultimately, 76,158 high-quality SNP loci were obtained from the 156 germplasm samples, which were used for subsequent analyses.

### 4.4. Principal Component Analysis

Principal Component Analysis (PCA) was performed on the obtained genotype data to reveal the genetic variation among different soybean germplasm. PCA was conducted using R (version 4.4.0) and PLINK (version 1.90b7.2) software, based on high-quality SNP loci (a total of 76,158 SNP loci) [30]. The results of the PCA were visualized by plotting the principal component scores (e.g., PC1 vs. PC2), which allowed for the assessment of genetic distances and population structure among the germplasm samples.

### 4.5. PVE

This study calculated the Phenotypic Variance Explained (PVE) using multiple models to assess the contribution of genetic effects to phenotypic variation. Specifically, PVE was calculated as the ratio of genetic variance to total phenotypic variance, as follows:

$$PVE=\frac{\mathrm{Genetic}\;\mathrm{Effect}\;\mathrm{Variance}}{\mathrm{Phenotype}\;\mathrm{Variance}}$$

The following models were used in the calculation process: GBLUP, BayesA, BayesB, BayesC, BL, BRR, Kernel Ridge, Linear Regression, PLS Regression, Ridge Regression, SVR Linear, and SVR Poly. Each model was fitted to the genotype data to extract genetic variance, and the total phenotypic variance was calculated [31,32]. By comparing these models, the study provided a comprehensive assessment of the contribution of genetic factors to phenotypic variation and determined the corresponding PVE values.

### 4.6. GWAS Analysis and Candidate Gene Annotation

To identify SNP loci associated with soybean cold tolerance, this study employed three GWAS methods: Fast3VmrMLM, fastGWA, and the FarmCPU method in rMVP [15,33,34]. Each method was corrected for population structure using the first three principal components. Manhattan plots generated using the R package CMPlot (version 4.4.0) were used to visualize the GWAS results. Subsequently, the number of overlapping statistically significant markers (*p* < 0.05) for each method was visualized using Venn diagrams to evaluate the differences between the algorithms [35]. Based on the GWAS results, genes near the significant markers were searched, and to verify the reliability of the Gene Ontology (GO) analysis, the range of candidate genes was narrowed. GO enrichment analysis of these candidate genes was performed using TBtools (version 2.376) [36].

### 4.7. GWAS-GS

In this study, genome selection (GS) analysis was first performed using all SNP markers. The analysis utilized SNP data across the entire soybean genome to comprehensively assess the ability of markers to predict cold tolerance phenotypes at the genome-wide level. In the GS analysis, the first six models (GBLUP, BayesA, BayesB, BayesC, BL, and BRR) were implemented using the BGLR package in R (version 4.4.0) [31], while the subsequent models (Kernel Ridge, Linear Regression, PLS Regression, Ridge Regression, SVR Linear, and SVR Poly) were analyzed using the sklearn library in Python (version 3.10) [32,37]. All models were evaluated using 5-fold cross-validation and repeated 50 times to ensure the stability and reliability of the prediction results.

To further assess the prediction ability of the SNP markers selected by GWAS, SNP markers significantly associated with soybean cold tolerance phenotypes (*p* < 0.05) were chosen based on the GWAS results. Then, these significant SNP markers were used for genomic selection, ranked by *p* value. To investigate the effect of SNP density on prediction, five density gradients (500, 1000, 5000, 10,000, and 50,000 top-ranked SNPs) were evaluated. All models were assessed using five-fold cross-validation, and each model was repeated 50 times to ensure the accuracy and stability of the predictions. Prediction accuracy was evaluated using the Pearson correlation coefficient (Pearson’s r) between the predicted and observed phenotypic values.

## 5. Conclusions

This study successfully improved the prediction accuracy of soybean cold tolerance traits by integrating Genome-Wide Association Studies (GWAS) and Genome Selection (GS) methods. The results show that GWAS methods, by identifying significant SNP markers associated with cold tolerance traits, effectively enhanced the performance of genomic selection models (especially BayesA and BayesB) in predicting soybean cold tolerance traits. When comparing the markers selected by different GWAS methods (such as Fast3VmrMLM, fastGWA, and FarmCPU) with all markers, the findings indicate that GWAS selection significantly improved the prediction accuracy of genomic selection models, particularly when larger datasets were used. Furthermore, this study also demonstrates the significant advantage of Bayesian regression models in genomic selection for soybean cold tolerance, as they effectively capture genetic variation and improve prediction accuracy. Compared to traditional genomic selection methods (such as GBLUP), the Bayes series models were more effective in predicting cold tolerance traits. However, the study also has certain limitations, including reliance on phenotypic data, limitations in marker density, and the impact of environmental factors on prediction accuracy. Future research could incorporate more environmental data, cold tolerance traits from different growth stages, and higher-density genomic data to further enhance the prediction accuracy of soybean cold tolerance traits. Overall, the integration of GWAS and GS methods provides a new technological framework for the precise breeding of soybean cold tolerance traits and offers theoretical support for the development and molecular improvement of cold-tolerant soybean varieties. With the advancement of genomics and increasing data volume, genomic selection is expected to be more widely applied in the breeding of soybean and other crops, driving sustainable agricultural production.

## Figures and Tables

**Table 1:** Significant SNP markers and associated candidate genes for soybean cold tolerance identified by GWAS. This table lists the significant SNPs and their corresponding candidate genes on various chromosomes. Candidate genes were identified based on physical proximity, utilizing a conservative threshold of ±2 kb flanking each significant SNP to ensure high-confidence assignment. The columns provided include the chromosome number (chr), the physical coordinates (start and end), the candidate gene identifier (gene_name), the specific SNP location (snp_pos), and the GWAS model detecting the marker (gwas_marker).

Chr	Start	End	Gene_Name	Snp_Pos	Gwas_Marker
Gm03	6679636	6694991	*Glyma.03G052151*	6685414	Gm03_6685414
Gm08	46134008	46143597	*Glyma.08G318000*	46144933	Gm08_46144933
Gm11	6634544	6638369	*Glyma.11G087900*	6634392	Gm11_6634392
Gm11	6685236	6691789	*Glyma.11G088400*	6686188	Gm11_6686188
Gm11	6706992	6712243	*Glyma.11G088600*	6707427	Gm11_6707427
Gm11	6766509	6768581	*Glyma.11G089200*	6768477	Gm11_6768477
Gm11	6790735	6795318	*Glyma.11G089500*	6791972	Gm11_6791972
Gm11	6824997	6831218	*Glyma.11G090100*	6832069	Gm11_6832069
Gm11	6852742	6856435	*Glyma.11G090200*	6857935	Gm11_6857935
Gm11	6885814	6891986	*Glyma.11G090900*	6888005	Gm11_6888005
Gm15	17240737	17244374	*Glyma.15G179300*	17239798	Gm15_17239798
Gm19	48536543	48545780	*Glyma.19G196700*	48542345	Gm19_48542345

## Data Availability

The original contributions presented in this study are included in the article/Supplementary Materials. Further inquiries can be directed to the corresponding authors.

## Supplementary Materials

The following supporting information can be downloaded at https://www.mdpi.com/article/10.3390/ijms27010165/s1.
