# Supplementary material for: Integrating Genome-Wide Association Study (GWAS) and Marker-Assisted Selection for Enhanced Predictive Performance of Soybean Cold Tolerance
Source: Int J Mol Sci. 2025 Dec 23;27(1):165. doi: 10.3390/ijms27010165 (PMC12786165; doi:10.3390/ijms27010165)
Supplement: Supplementary file 1 [file ijms-27-00165-s001.zip › ijms-4033428-supplementary.pdf]

Table S1: Summary of Soybean Germplasm Resources. This table lists the soybean germplasm resources evaluated for cold tolerance, with the corresponding identification numbers and names for both European and Chinese germplasm. The "Number" column refers to the identification number for each germplasm, while the "Name (Europe)" and "Name (China)" columns provide the names of the soybean varieties from Europe and China, respectively.

Number	Name (Europe)	Number	Name (China)
A1	CH22172 / OBELIX	C1	Dongnong54
A2	CH21912 / PROTEIX	C2	Dongnong53
A3	CH22138 / AMANDINE	C3	Dongnong58
A4	CH22177 / GALICE	C4	Dongnong50
A5	CH22315 / MARQUISE	C5	Dongnong52
A6	CH21265 / ORION	C6	Heihe43
A7	CH22429	C7	Heihe36
A8	CH22015 / CASTETIS	C8	Heihe44
A9	CH50111	C9	Heihe49
A10	CH50051 / PACO	C10	Dongnong55
A11	Atlanta	C11	Heinong68
A12	Mavka	C12	Hefeng51
A13	ADA TD	C13	Dongnong51
A14	CRISTINA TV	C14	Heihe45
A15	FELIX	C15	Heihe52
A16	MIRUNA	C16	Heinong51
A17	ERICA	C17	Heinong61
A18	PEPITA	C18	Heinong64
A19	AMMA	C19	Heinong69
A20	Ananda	C20	Jiyu95
A21	BAHIA	C21	KeshangNo.1
A22	Prana	C22	DengkeNo.1
A23	ADONAI	C23	Dengke-2
A24	Avatar	C24	Mengdou30
A25	BLANCAS	C25	Suinong22
A26	Buenos	C26	Suinong23
A27	Guru	C27	Suinong24
A28	ES Senator	C28	Suinong26
A29	ES Gladiator	C29	Suinong27
A30	ES Tenor	C30	Suinong29
A31	ES Indicator	C31	Suinong32
A32	ES Mediator	C32	Suinong33
A33	GK MEDAL	C33	Suinong34
A34	GK SPIRIT	C34	Suinong35
A35	PANNONIA KINCSE	C35	Hefeng35
A36	Khutorianochka	C36	Henong61
A37	Oriana	C37	Heihe39
A38	TriaDa	C38	Hefeng48
A39	Vezha	C39	Hefeng55
A40	NS Kaća	C40	Henong60
A41	Favorit	C41	Heinong52
A42	Galina	C42	Hefeng39
A43	NS Atlas	C43	Hefeng56
A44	NS Maximus	C44	Henong59
A45	NS Mercury	C45	Hefeng50
A46	NS Princeza	C46	Hefeng57

Number	Name (Europe)	Number	Name (China)
A48	NS-L-201458	C47	Henong62
A49	NS-L-401088	C48	Henong58
A50	NS-L-401145	C49	Kengnong36
A51	NS-L-401156	C50	Kengbao No.1
A52	NS-L-401157	C51	Kengfeng16
A53	NS-L-501012	C52	Heinong63
A54	Tajfun	C53	Heihe48
A55	Valjevka	C54	Heihe51
A56	NS Fantast	C55	Heinong53
A57	NS HOGAR	C56	Fengshou22
A58	NS Kraljica	C57	Fengshou25
A59	NS Zita	C58	Kenfeng20
A60	Sava	C59	Beifeng16
A61	Trijumf	C60	Kenfeng17
A62	Venera	C61	Fengshou26
A63	Ventis	C62	Hefeng53
A64	Victoria	C63	Heihe No.5
A65	AUGUSTA	C64	Beidou30
A66	Amadea	C65	Beidou44
A67	Antonia	C66	Fengshou27
A68	Abelina	C67	Kedou28
A69	Albenga	C68	Beidou40
A70	Alexa	C69	华疆 1 号
A71	Ancona	C70	Fengshou12
A72	Angelica	C71	Hefeng49
A73	Regina	C72	Hefeng54
A74	Christine	C73	Fengshou11
A75	GL Hermine	C74	Fengshou13
A76	Josefine	C75	Beifeng17
A78	SM SR16050	C76	Beifeng No.4
A79	smsr 17046	C77	Kenfeng13
		C78	Suinong No.7
		C79	Heinong22
